# The Voluntary Hospitals and Legacy Duty

**Published:** 1913-02-22

**Authors:** 


### THE VOLUNTARY HOSPITALS AND LEGACY DUTY.

### Away with Legacy Duty on Hospital Legacies!

In our issue of January 25, p. 453, we printed certain facts which showed that the Treasury have steadily neglected the interests of voluntary hospitals in recent years. Two memorials have been addressed to the Chancellor of the Exchequer on the subject of legacy duty on hospital legacies, and the following reply just received proves to demonstration that unless the hospitals, like every other interest nowadays, organise themselves into a political force, quite apart from party, they may be irretrievably damaged or destroyed at the will of any Cabinet which continuously puts the interests of party before the interests and welfare of the people of these islands. Mr. H. P. Hamilton writes in the same old plausible official way to Mr. Conrad W. Thies as Honorary Secretary to the British Hospitals Association:—

"The Chancellor of the Exchequer has now given his most careful consideration to your letter of the 15th ultimo, enclosing a memorial on behalf of the British Hospitals Association, asking that hospitals should be relieved from payment of legacy duty in respect of legacies bequeathed to them. Having regard, however, to the very considerable loss of revenue to the Exchequer which the proposal involves and to the very serious demands of a like nature which would inevitably follow any such concession, Mr. Lloyd George regrets that he cannot see his way to submit to Parliament legislation with the object of exempting hospitals from the payment of this duty. I am, moreover, to point out that it is always open to a testator to bequeath a legacy to a hospital 'free of duty,' and in this way to shift the incidence of duty from the hospital to the residuary legatee."

The first question which arises on this routine letter, to our mind, is, Will the voluntary hospitals stand this further and most inequitable, not to say ungenerous, disregard of the enormous claims they have to-day upon the Chancellor of the Exchequer owing to the services they are at present rendering to the British Treasury through their action in regard to the Insurance Act? Were it not for the free medical benefit which the voluntary hospitals are providing for insured persons, Mr. Lloyd George's Insurance Act could not work, and must be suspended until, by further legislation, it had been made into a statesmanlike and just measure. Fortunately, the remedy, which has never yet failed to be effective when applied to the present Cabinet, is ready to hand. Let every voluntary hospital and kindred charity throughout the country unite their forces and enter upon a vigorous campaign, which must compel Parliament as a mere act of justice to grant this—to the British Treasury—relatively immaterial remission of legacy duty for the benefit of voluntary hospitals. These institutions are equitably entitled to such remission without further delay. It is at least satisfactory to have so plain an intimation from Mr. Lloyd George that the hospitals need expect no help in this matter unless they prove themselves strong enough to enforce their claims through the Parliamentary representatives of the district where each hospital is situated, as they can readily do in present circumstances, without difficulty or any prospect of failure. Energetic, continuous, untiring pressure during the next few months would relieve the voluntary hospitals from legacy duty with practical certainty. Perhaps Scotland, failing London, Wales, and England, may lead the way. This is a business question, and as such should be taken up at once and fought to a finish, for victory is then assured.

